# Revealing the Dysfunction of Schematic Facial-Expression Processing in Schizophrenia: A Comparative Study of Different References

**DOI:** 10.3389/fnins.2017.00314

**Published:** 2017-05-31

**Authors:** Shenglin She, Haijing Li, Yuping Ning, Jianjuan Ren, Zhangying Wu, Rongcheng Huang, Jingping Zhao, Qian Wang, Yingjun Zheng

**Affiliations:** ^1^Department of General Psychiatry, The Affiliated Brain Hospital of Guangzhou Medical University (Guangzhou Huiai Hospital)Guangzhou, China; ^2^National Clinical Research Center for Mental Disorders, Mental Health Institute, The Second Xiangya Hospital of Central South UniversityChangsha, China; ^3^Beijing Key Laboratory of Epilepsy, Department of Functional Neurosurgery, Epilepsy Center, Sanbo Brain Hospital, Capital Medical UniversityBeijing, China

**Keywords:** schizophrenia, facial expression, visual mismatch negativity (vMMN), average reference (AVE), reference electrode standardization technique (REST)

## Abstract

The use of event-related potential (ERP) recording technology during perceptual and cognitive processing has been studied in order to develop objective diagnostic indexes for people with neuropsychiatric disorders. For example, patients with schizophrenia exhibit consistent abnormalities in face-evoked early components of ERPs and mismatch negativities (MMNs). In most studies, the choice of reference has been the average reference (AVE), but whether this is the most suitable choice is still unknown. The aim of this study was to systematically compare the AVE and reference electrode standardization technique (REST) methods for assessing expressional face-evoked early visual ERPs and visual MMNs (vMMNs) in patients with schizophrenia and healthy controls. The results showed that both the AVE and REST methods could: (1) obtain primary visual-evoked ERPs in the two groups, (2) reveal the neutral and emotional expression discrimination deficit of the P1 component in the patients, which was normal in the healthy controls, (3) reflect reductions of happy vMMNs in the patients compared to the healthy controls, and (4) show right-dominant sad vMMNs only in the patients. On the other hand, compared to the energy distributions of the AVE-obtained potentials, those of REST-obtained early visual ERPs and vMMNs were more concentrated around the temporo-occipital areas. Furthermore, only the REST-obtained vMMNs revealed a significant difference between happy and sad mismatch stimuli in patients with schizophrenia. These results demonstrate that REST technology might provide new insights into neurophysiological factors associated with neuropsychiatric disorders.

## Introduction

Schizophrenia is a psychiatric disorder that is associated with various clinical symptoms such as auditory hallucinations, paranoid delusional thoughts, disorganized thinking, and disturbances of self (Diagnostic and Statistical Manual of Mental Disorders, Fourth Edition) (American Psychiatry Association, 1994; Onitsuka et al., 2013). One of the cognitive dysfunctions in people with schizophrenia is facial processing at both the behavioral (Chen, 2011; Maher et al., 2015, 2016) and physiological levels (Herrmann et al., 2004; Onitsuka et al., 2006; Tsunoda et al., 2012; Maher et al., 2016; Zheng et al., 2016). Many recent studies have shown that people with diagnosis of schizophrenia often exhibit impairments in facial expression recognition, which are suggested to be related to poor social functioning (Michalopoulou et al., 2008; Kohler et al., 2009; Mendoza et al., 2011; Csukly et al., 2013; McCleery et al., 2015). The perception of facial expressions, which provides a fundamental emotional analysis of the mental intention of a person, is found to be abnormal in people with schizophrenia (for review see McCleery et al., 2015). Previous studies have mainly focused on the recognition or memory of facial expressions in patients with schizophrenia (Guillaume et al., 2012). However, a recent study reveals that the visual mismatch negativities (vMMNs) evoked by changes of facial expressions could also be abnormal in schizophrenia (Csukly et al., 2013).

Related to automatic processing, MMN is defined as the difference between the potentials evoked by deviant (infrequent) and standard (frequent) stimuli (Näätänen et al., 2004). Accumulating evidence has suggested that the variation of not only the low-level features of visual stimuli such as color, motion, or spatial frequency, but also high-level facial expressions could effectively evoke vMMN (Pazo-Alvarez et al., 2003; Susac et al., 2004; Zhao and Li, 2006; Czigler, 2007; Astikainen and Hietanen, 2009; Li et al., 2012). Previous studies have shown that a right-posterior facial expression vMMN elicited by sad and happy faces rather than neutral ones (Zhao and Li, 2006) was possibly generated from the combination of occipital, temporal, and frontal areas (Kimura et al., 2012; Stefanics et al., 2012).

Recently, new studies investigating vMMN in patients with psychiatric disorders have been conducted (for review see Kremláček et al., 2016). For example, a previous study has shown that deviant motion direction could elicit a reduction of vMMN signals in patients with schizophrenia, indicating the impairment of early processing of visual information (Urban et al., 2008). In an investigation that is particularly relevant to the current study, Csukly et al. (2013) studied the deficit in vMMN toward irregular expression in patients with schizophrenia and found that, compared to healthy people, neither happy nor fearful faces elicited any mismatch responses in the patients with schizophrenia. This indicates that patients with schizophrenia have insufficient automatic processing of emotions, which correlates with emotional recognition deficits. As patients with schizophrenia frequently show abnormal processing of emotional expressions, it should be emphasized on the neurophysiological evidences in schizophrenic population.

Technologically, many of the clinical studies prefer lower number of recording electrodes, which raises the question of whether the choice of electroencephalogram (EEG) reference electrodes could affect the results. Whether the reference methods could significantly affect the EEG are still under debate. The previous facial expression vMMN study (Csukly et al., 2013) used averaged reference (AVE) method. However, accumulating evidences suggest that a neutral reference such as the reference electrode standardization technique (REST) should be used in EEG studies (Yao, 2001; Zhai and Yao, 2004; Liu et al., 2015; Chella et al., 2016). Although the previous vMMN studies with AVE are broadly accepted, a recent study claims that REST shows a more reliable scalp signal reconstruction with low-density montage than AVE (Liu et al., 2015). Therefore, it should be essential to find whether some new insight could be gained when REST is adopted. Thus, in the current study, AVE and REST methods were both conducted to study expressional face-evoked early visual event-related potentials (ERPs) and vMMNs in schizophrenic patients and healthy controls. Faces with sad and happy expressions were used as deviant stimuli, and faces with neutral expressions as standard stimuli under situations (Chang et al., 2010). To avoid the variance inherent with images of actual faces, schematic emotional faces were used, because faces that are formed using simple lines are sufficient to arouse face-specific activity (Sagiv and Bentin, 2001; Wright et al., 2002). It is hypothesized that; (1) there is early visual dysfunction in processing emotional faces, (2) a reduced vMMN will occur in patients with schizophrenia in comparison with healthy participants, and (3) REST-obtained potential might be better in revealing the neurophysiological differences between patients with schizophrenia and healthy participants.

## Methods

### Participants

Twenty-three patients with schizophrenia (11 females, mean age 32.3 ± 11.1 years old) and 23 age-matched healthy controls (11 females, mean age 32.6 ± 11.3 years old) participated in the current study. All the participants had normal or corrected-to-normal binocular visual acuity. They could read all “E” orientations correctly at line 5.0/1.0 in a visual acuity chart (from 3 meters) using each eye. Each patient was diagnosed with schizophrenia in accordance with the Diagnostic and Statistical Manual of Mental Disorders, Fourth Edition. None of them were first-episode patients, and the time relative to their first diagnosis was at least 2 years. None of the included patients had a history of severe medical disorder or severe neurological disorder. An experienced psychiatrist or psychologist evaluated clinical symptoms using the Positive and Negative Syndrome Scale (PANSS) (Kay et al., 1987). The basic demographic and descriptive characteristics of the participants are showed in Table 1.

**Table 1:** Basic demographic and descriptive characteristics in both groups.

	**Patients with schizophrenia (*n* = 23)**	**Healthy control subjects (*n* = 23)**	***P***
Gender (male/female)	12/11	12/11	1.0^a^
Education (years)	12.9 (2.6)	12.5 (3.5)	0.707^b^
Average family income (RMB/per year)	5079.2 (3724.9)	6291.67 (3473.2)	0.249^b^
Handedness (right/left)	23/0	23/0	
Schizophrenia subtypes: Paranoid/Undifferentiated	16/7	N/A	
Duration of illness (years)	8.7 (6.3)	N/A	
PANSS total	52.4 (12.4)	32.3 (1.5)	0.000^b^
PANSS positive symptoms	13.3 (5.4)	7.4 (0.7)	0.000^b^
PANSS negative symptoms	11.2 (4.2)	7.2 (0.4)	0.000^b^
PANSS general symptoms	27.9 (6.2)	17.7 (1.2)	0.000^b^
Antipsychotic medication (Atypical/Typical)	21/2	N/A	
Chlorpromazine equivalent (mg)	556.5 (350.2)	N/A	
PSP	60.5 (9.9)	89.1 (4.3)	0.000^b^

a *Binomial*.

b *T-test*.

*PANSS, Positive and Negative Syndrome Scale*.

No history of any serious mental illnesses was reported among the healthy volunteers, and they did not use any medications that affected the central nervous system. No neurological illness or brain injury, addiction, or visual impairment existed in either of the groups. The Institutional Review Board of Guangzhou Brain Hospital approved all the experimental procedures. All participants received payments and provided written informed consent for their participation.

### Stimuli and procedure

**Figure 1:**
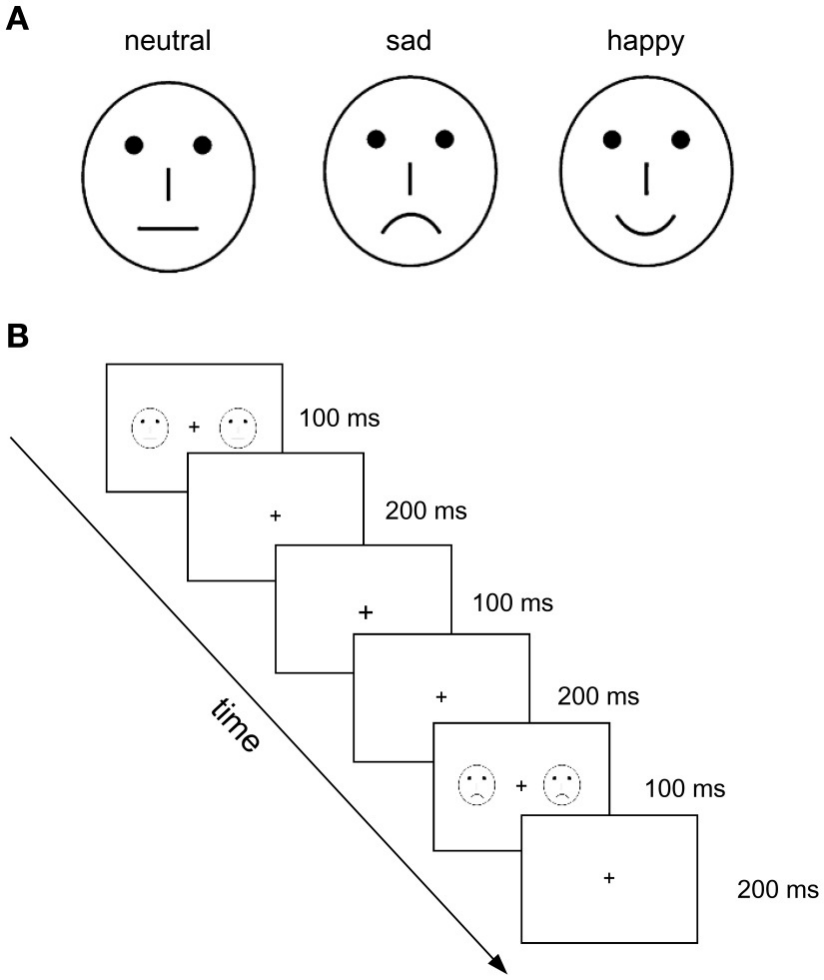
Illustration of experimental stimuli and procedure used in the present study. **(A)** Examples of schematic face stimuli that contain three facial expressions: neutral, sad, and happy. **(B)** Illustration of the experimental procedure. The neutral faces served as standard stimuli and happy and sad faces as deviant stimuli. The task was to judge the change in size of the fixation cross (“+”) in the visual center.

As presented in a previous study conducted by this research group (Xu et al., 2013), to reduce the effect of low-level features, 54 different schematic faces with neutral, sad, and happy expressions were used, and 18 individual schematic faces were included for each stimulus type (see an example in Figure 1A). Modulated by changing the shape of and the distance between the facial features, each type of stimulus included 18 models. The visual stimuli were presented on both sides of the fixation, and the duration of exposure was 100 ms, with a 500 ms inter-stimulus interval, and a visual angle of 3.68° × 3.42°.

The three-stimulus oddball paradigm was used. Figure 1B shows the experimental procedure. The neutral faces served as standard stimuli, and happy and sad faces as deviant stimuli. To establish a sensory memory pattern, 10 standard stimuli (neutral faces) were presented at the beginning of a stimulus sequence, and with no less than two standards between consecutive deviants. The participants were asked to focus on the fixation crosses with a changed size, which were always presented without faces to avoid motor-generated artifacts. Four blocks were conducted with 250 trials for each (standard: 200 neutral faces; deviant: 25 happy and 25 sad faces). The task performed by the participants was to judge the change in size of the fixation cross (“+”) in the visual center. Practice trials were conducted before the test trials. There was a 1-min break between blocks.

### Electrophysiological recording

EEG was recorded continuously by a set of 16 Ag/AgCl electrodes placed according to the 10/20 system, including F3, Fz, F4, C3, Cz, C4, P7, P3, Pz, P4, P8, PO7, PO8, O1, Oz, and O2. Electrooculography (EOG) was recorded via electrodes placed on the bilateral external canthi and the left infraorbital and supraorbital areas to monitor for eye movements and blinks. Both EEG and EOG were sampled at 1,000 Hz, with a 0.1–100 Hz band pass using a Neuroscan NuAmps digital amplifiers system (Neuroscan Labs, El Paso, TX). The tip of the nose was used as reference during recording. Impedances of all electrodes were kept below 5 kΩ.

### Data analyses

The pre-processing of the electrophysiological data was conducted by the functions of the EEGLAB toolbox (Delorme and Makeig, 2004) in MATLAB environment. Both average reference (AVE) and approximate zero reference (REST) (Yao, 2001) were conducted off-line to generate two long-term EEGs. The AVE was conducted by the *reref* function from EEGLAB toolbox (Delorme and Makeig, 2004) and the REST was conducted by the *rest_refer* function from www.neuro.uestc.edu.cn/rest. These long-term EEGs of each electrode were firstly filtered by a band pass filter (0.5 - 40 Hz) and then segmented into epochs from -100 to 500 ms around the onset. The baseline correction was conducted within the time window of -100 to 0 ms. The epochs that contained more than ±150 μV EOG potential were rejected as artifacts. The rest of the epochs were then averaged and low-pass filtered (cut-off frequency = 15 Hz) to obtain two groups of ERPs for AVE and REST methods, respectively. The amplitudes of early visual ERP components (P1 and N170) were analyzed to compare the primary sensory processing in the two groups. The vMMNs were obtained by subtracting ERPs to standard stimuli (neutral faces) from ERPs to deviant stimuli (sad or happy faces) for each facial expression.

Statistical analyses were performed with IBM SPSS Statistics 20 (SPSS Inc., Chicago, Illinois 60606). Analyses of variance (ANOVAs), *post-hoc* tests and *t*-tests were conducted. *P* values were corrected by *Bonferoni* adjustment to avoid multiple comparisons. The null-hypothesis rejection level was set at 0.05.

## Results

The results of behavioral data showed that, for healthy controls, the mean reaction time and accuracy rate were 368 ms (*SD* = 80 ms) and 96.6 % (*SD* = 3.5%), respectively, while for schizophrenic patients, the mean reaction time and accuracy rate were 375 ms (*SD* = 75 ms) and 96.2 % (*SD* = 4.2%), respectively. Independent *t*-tests found no significant difference between the two groups (both *p* > 0.05).

### Face evoked P1 and N170 components

After the artifact rejection, the mean numbers of epochs under neutral face conditions were 220.92 (*SD* = 11.61) and 221.88 (*SD* = 11.96), sad face conditions were 86.24 (*SD* = 4.21) and 85.92 (*SD* = 4.67), and happy face conditions were 85.24 (*SD* = 5.43) and 85.88(*SD* = 5.15), in control and patient groups, respectively. Independent *t*-tests showed no significant differences between epochs of the two groups under any of the conditions.

**Figure 2:**
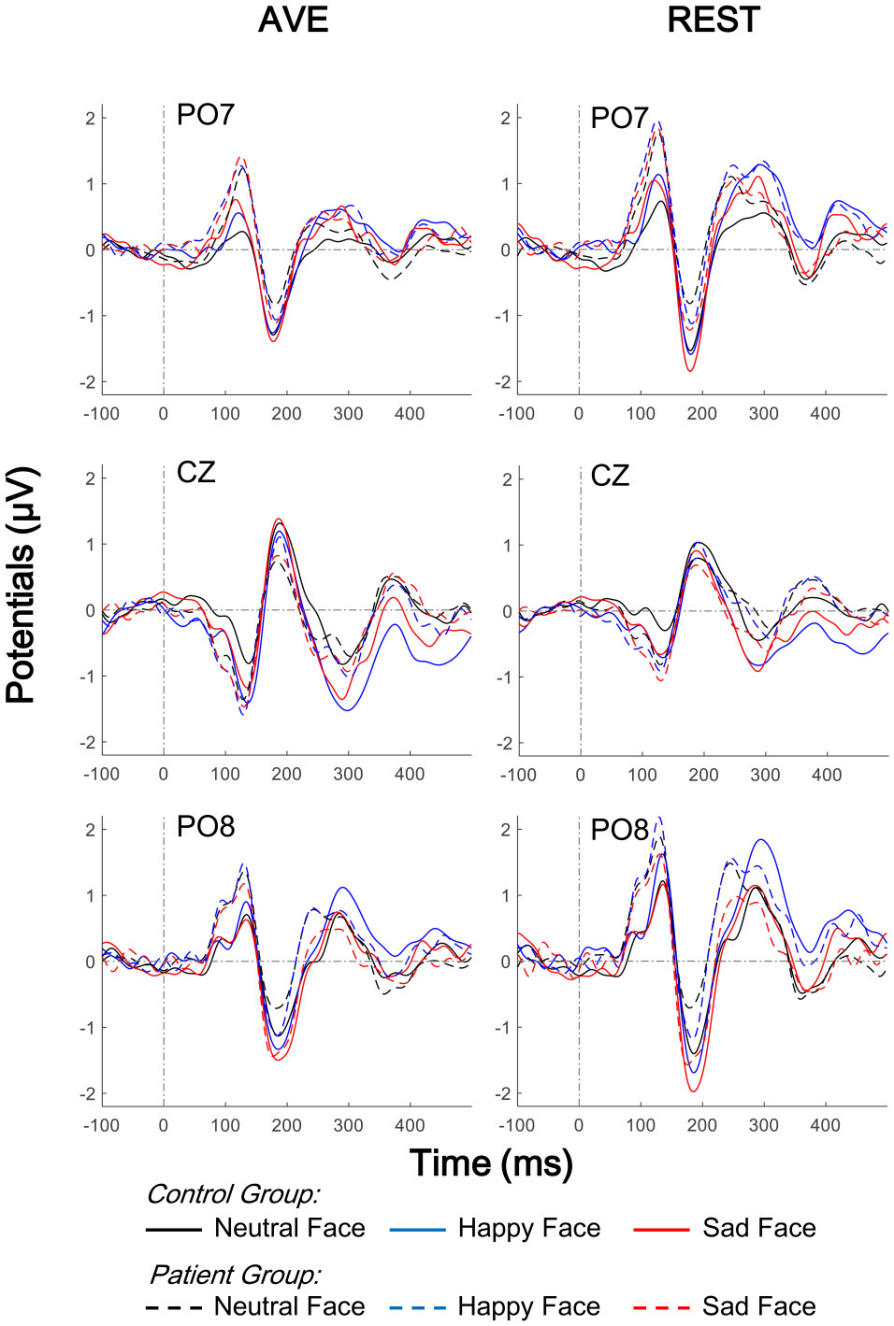
Comparison of face evoked ERPs using two reference-modes (AVE and REST). Waveforms of temporo-occipital electrodes (PO7, PO8) and central electrode (CZ) ERPs evoked using three stimulus conditions (neutral, happy, and sad facial expressions) in both control and patient groups are also compared. Solid lines, control group; Broken lines, patient group; AVE, average reference; REST, reference electrode standardization technique; ERP, event-related potential.

**Figure 3:**
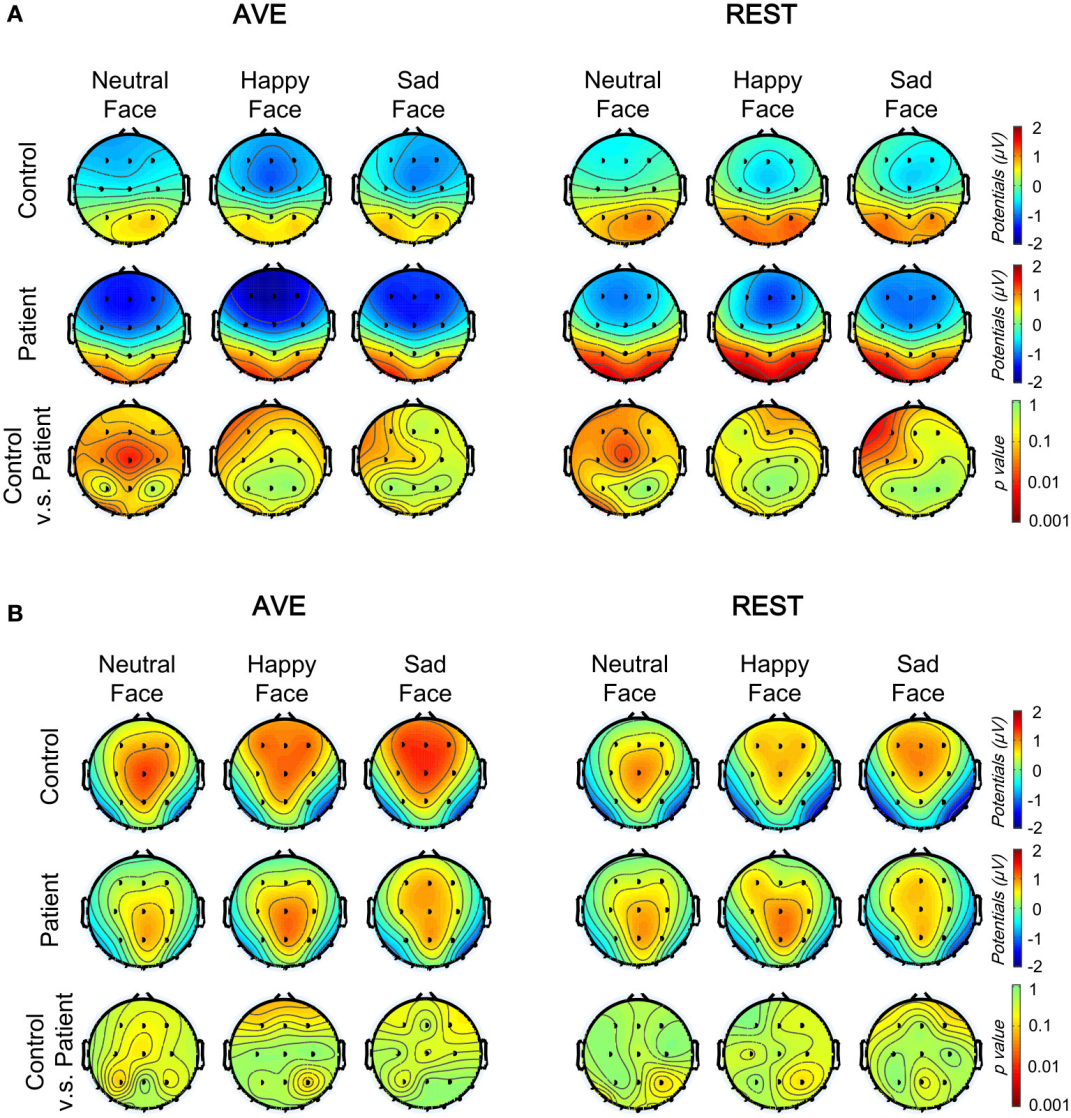
Comparison of face evoked topographic distributions using two reference-modes (AVE and REST). **(A)** The topographic distributions of the P1 mean amplitudes (110–130 ms) under three conditions in control (top) and patient (middle) groups, and between group significance analyses (bottom, with *Bonferoni* correction). **(B)** The topographic distributions of the N170 mean amplitudes (175–195 ms) under three conditions in control (top) and patient (middle) groups, and between group significance analyses (bottom, with Bonferoni correction). AVE, average reference; REST, reference electrode standardization technique.

Figure 2 shows the grand average ERPs at the temporo-occipital electrodes (PO7 and PO8) and central electrode (CZ) evoked by deviant and standard faces in both groups. The primary visual processing components including P1 and N170 were observed in both schizophrenic patients and healthy controls. After comparing the AVE and REST results, the REST gave higher evoked potentials around the temporo-occipital electrodes (PO7 and PO8) and lower evoked potentials in CZ. This observation was further confirmed in topographic analyses. The results showed that, compared to AVE-obtained components, the absolute amplitudes of REST-obtained P1 component (110 to 130 ms, Figure 3A) were higher around the temporo-occipital areas (more positive) and lower around the central areas (less negative), while those of REST-obtained N170 component (175 to 195 ms, Figure 3B) were also higher around the temporo-occipital areas (more negative) and lower around the central areas (less positive), under all conditions. These results indicated that REST-obtained visual evoked ERPs gave significant temporo-occipital distributions in both schizophrenic patients and healthy controls.

In order to evaluate the group and stimulation effects on P1 and N170 potentials with different reference technologies, 2 (Group: patient and control) × 3 (Facial expression: neutral, happy, and sad) × 2 (Reference type: AVE and REST) three-way mixed-measured ANOVAs were conducted, respectively. The P1 amplitude was defined as the averaged amplitude from 110 to 130 ms after stimulus onset, while the N170 amplitude was defined as the averaged amplitude from 175 to 195 ms. Three electrodes, PO7, PO8, and CZ were involved in the analysis, representing the temporo-occipital and central areas, respectively.

**Figure 4:**
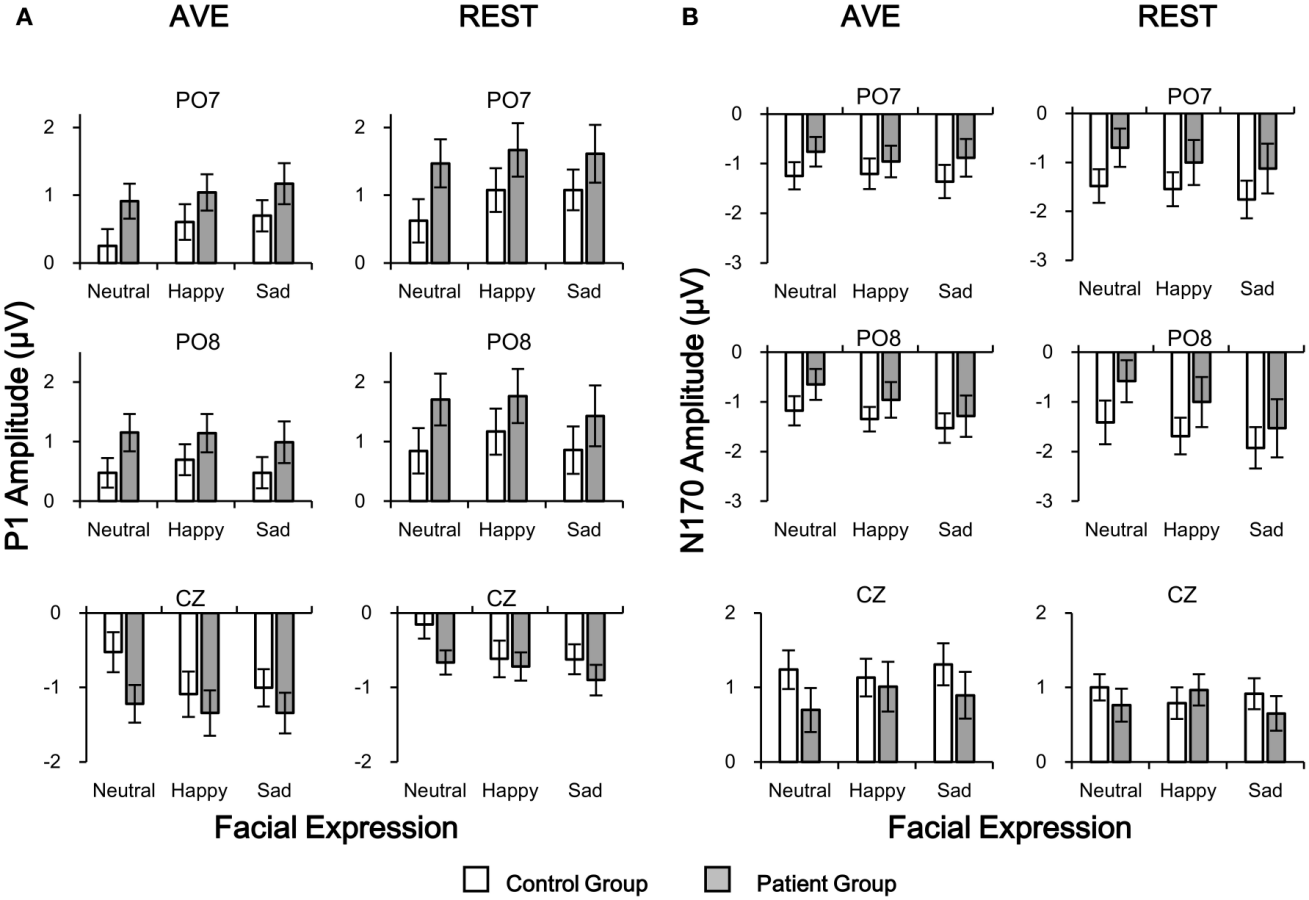
Comparisons of P1 **(A)** and N170 **(B)** amplitudes in patient and control groups in temporo-occipital electrodes (PO7 and PO8) and central electrode (CZ) using two reference-modes (AVE and REST). White bars, control group; Gray bars, patient group. AVE, average reference; REST, reference electrode standardization technique.

For P1 amplitudes, neither main effect of Group nor Reference type was significant (all *p* > 0.05). Significant Facial expression effect was found in central electrode [CZ: *F*_(1, 42)_ = 9.054, *p* = 0.004] but not in temporo-occipital electrodes [PO7: *F*_(1, 42)_ = 2.547, *p* = 0.118; PO8: *F*_(1, 42)_ = 0.195, *p* = 0.661]. No interaction effect was found (all *p* > 0.05). *Post-hoc* results with *Bonferoni* adjustment showed that the REST-obtained P1 amplitude was significantly more positive than the AVE-obtained ones in PO7 and PO8, and less negative in CZ (all *p* < 0.05) in both groups and all stimulus conditions (Figures 2, 4A). *Post-hoc* results also showed that in the control group, both AVE-obtained (CZ: neutral vs. happy, *p* = 0.001; neutral vs. sad, *p* = 0.004; happy vs. sad, *p* = 1.000; PO7 and PO8: no significance) and REST-obtained (CZ: neutral vs. happy, *p* = 0.008; neutral vs. sad, *p* = 0.002; happy vs. sad, *p* = 1.000; PO7 and PO8: no significance) P1 amplitude had significant difference between neutral and emotional expressions; while in the patient group, no significance between neutral and emotional expressions was found. No other significant *post-hoc* results were found. These results demonstrated that both the REST and AVE methods could reveal a central dominant face expression specific P1 component in healthy controls, which failed to distinguish neutral and emotional expressions in schizophrenic patients.

For N170 amplitudes, no significance of any main effect or interaction effect was found (all *p* > 0.05). *Post-hoc* results with *Bonferoni* adjustment showed that in the control group, the REST-obtained N170 amplitude was significantly more negative than the AVE-obtained ones in PO7 and PO8, and less positive in CZ under sad expression condition (all *p* < 0.05), but not under neutral and happy expression conditions (all *p* > 0.05) (Figures 2, 4B). These significances did not exist in patient group (all *p* > 0.05). No other significant *post-hoc* results were found.

In sum, these results demonstrated that, compared to the AVE-obtained visual processing ERPs, the energy of REST-obtained ERPs were more concentrated around temporo-occipital areas. Both REST and AVE methods could effectively discriminate early visual processing ERPs between healthy controls and schizophrenic patients.

### Visual mismatch negativity (vMMN)

**Figure 5:**
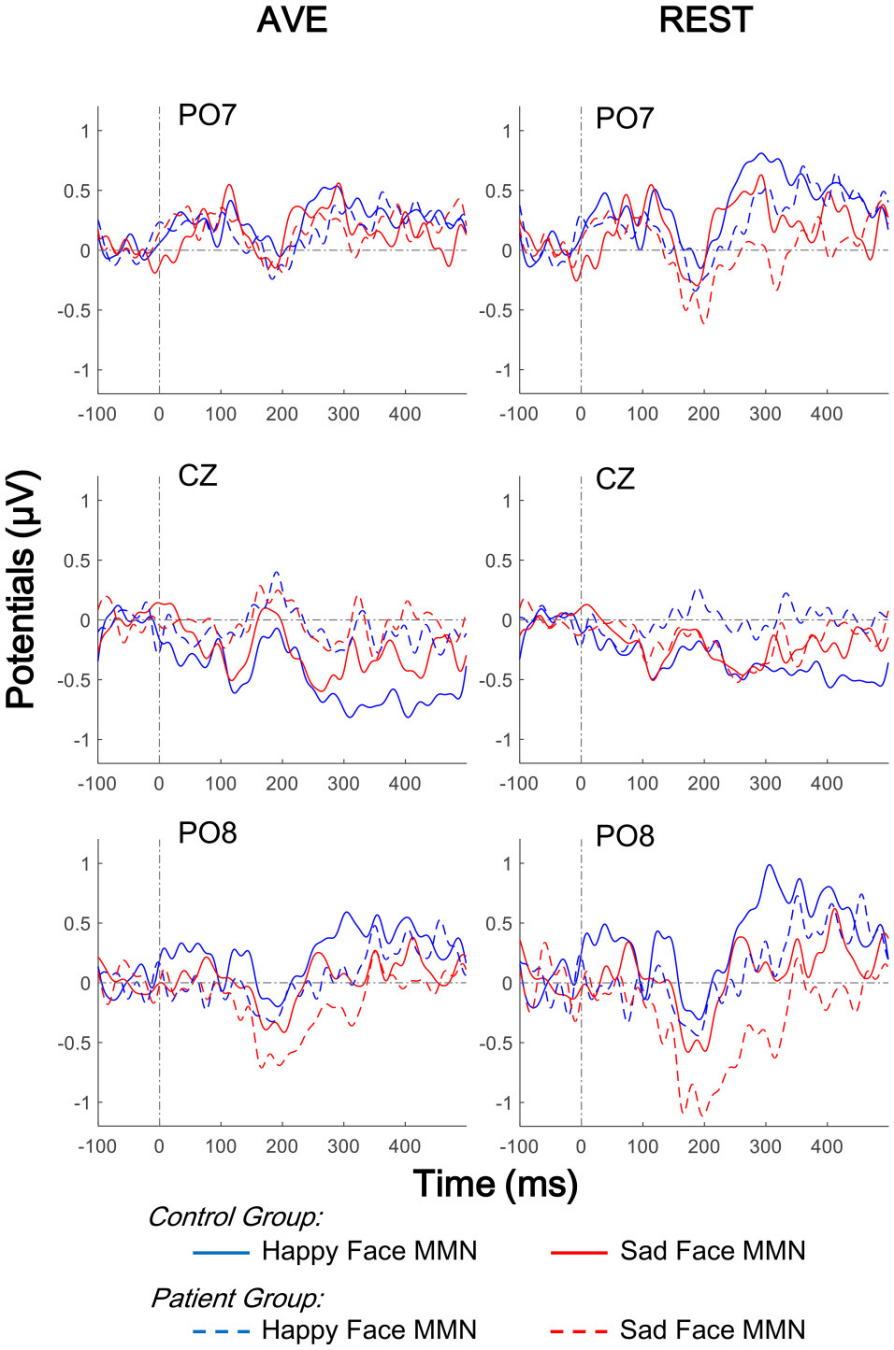
Comparison of face evoked vMMNs using two reference-modes (AVE and REST). Waveforms of temporo-occipital electrodes (PO7 and PO8) and central electrode (CZ) ERPs evoked using three stimulus conditions (neutral, happy, and sad facial expressions) in both control and patient groups are also compared. Solid lines, control group; Broken lines, patient group; AVE, average reference; REST, reference electrode standardization technique; vMMN, visual mismatch negativity.

**Figure 6:**
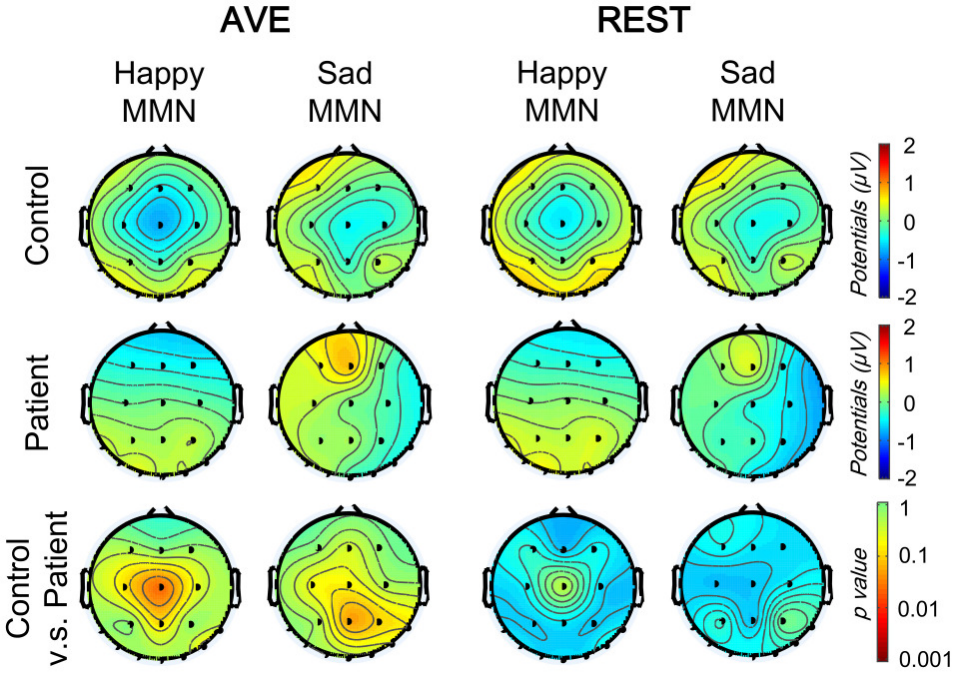
Comparison of vMMN topographic distributions using two reference-modes (AVE and REST). The topographic distributions of facial expression vMMNs (100–400 ms) under two conditions (happy and sad deviant) in control (top) and patient (middle) groups, and between group significance analyses (bottom, with Bonferoni correction). AVE, average reference; REST, reference electrode standardization technique.

Both happy and sad vMMN were calculated by subtracting ERPs of neutral faces from ERPs of sad and happy faces, respectively. Figure 5 shows the grand average vMMNs at the temporo-occipital electrodes (PO7 and PO8) and central electrode (CZ). Compared to the AVE-obtained vMMN, the REST-obtained vMMN gave higher evoked potentials around temporo-occipital electrodes (PO7 and PO8), and lower evoked potentials in CZ. The results of topographic analyses showed that for both reference methods, the distributions of vMMN were concentrated in the central areas in control group but not in patient group (100 to 450 ms, Figure 6). Furthermore, the differences between topographic distributions of controls and patients were also concentrated in the central areas (Figure 6, bottom panels).

To evaluate the group and stimulation effects on the vMMN potential with different reference technologies, 2 (Group: patient and control) × 2 (Mismatched facial expression: happy and sad) × 2 (Reference type: AVE and REST) three-way mixed-measured ANOVAs were conducted, respectively. Three electrodes, PO7, PO8, and CZ were involved in the analysis, representing the temporo-occipital and central areas, respectively.

The results showed that neither main effect of Mismatched facial expression nor Reference type was significant (all *p* > 0.05). Significant Group effect was found in central electrode [CZ: *F*_(1, 42)_ = 4.112, *p* = 0.049] but not in temporo-occipital electrodes [PO7: *F*_(1, 42)_ = 0.653, *p* = 0.424; PO8: *F*_(1, 42)_ = 3.052, *p* = 0.088]. No interaction effect was found (all *p* > 0.05). *Post-hoc* results with *Bonferoni* adjustment also showed that in patient group, the REST-obtained sad vMMN was significantly more negative that happy vMMN (CZ: *p* = 0.045; PO7 and PO8: no significance), but no significant difference between AVE-obtained sad and happy vMMN (CZ: *p* = 0.973; PO7 and PO8: no significance). *Post-hoc* results also showed that the happy vMMNs in patients were significantly lower than those in controls using both the AVE (CZ: *p* = 0.021; PO7 and PO8: no significance) and REST (CZ: *p* = 0.032; PO7 and PO8: no significance) methods. No other significant *post-hoc* results were found. These results demonstrated that both the REST and AVE methods could reveal significant differences between happy vMMNs in healthy controls and schizophrenic patients. Furthermore, only the REST-obtained vMMN revealed a significant difference between happy and sad mismatch stimuli in the patient group.

To evaluate the hemisphere effects on the vMMN potential of different stimulation and group, 2 [Group: patient and control) × 2 (Mismatched facial expression: happy and sad) × 2 (Hemisphere: Left (PO7) and Right (PO8)] three-way mixed-measured ANOVAs and *post-hoc* tests were conducted for AVE and REST, respectively. Interestingly, although no significant main effect or interaction effect was found, the *post-hoc* tests showed that the sad MMN in PO8 was significantly negative than that in PO7 in only the patient group (*p* = 0.018 for both AVE and REST). These results suggested a right hemisphere dominance of sad MMN in people with schizophrenia.

## Discussion

Face processing dysfunction has been widely explored in previous studies (Herrmann et al., 2004; Onitsuka et al., 2006; Chen, 2011; Tsunoda et al., 2012; Maher et al., 2015, 2016; Zheng et al., 2016); however, only a few have compared visual mismatch responses elicited by task-irrelevant facial expressions between healthy controls and patients with schizophrenia (Urban et al., 2008; Csukly et al., 2013). In the current study, although the performance in the detection task did not differ between the two groups of participants, both the AVE- and REST-obtained early visual ERPs and vMMN were different between the patient and control groups. Our hypotheses were clarified by the findings that; (1) early visual dysfunction in processing emotional faces existed in people with schizophrenia, (2) vMMN significantly reduced in people with schizophrenia in comparison to healthy participants, which had a right hemisphere dominance, and (3) generally, REST was as good as AVE in revealing the neurophysiological differences between people with schizophrenia and healthy people, while only the REST-obtain vMMN revealed a significant difference between happy and sad mismatch stimuli in schizophrenic patients.

### Expressional face-evoked P1 and vMMN dysfunction in schizophrenia

Generally, in the current study, both AVE and REST methods could effectively distinguish the expressional face-evoked P1 and vMMNs between healthy controls and schizophrenic patients, which were in accordance with previous studies of early visual ERPs (Herrmann et al., 2004; Onitsuka et al., 2006; Tsunoda et al., 2012; Maher et al., 2016; Zheng et al., 2016) and vMMNs (Urban et al., 2008; Csukly et al., 2013) using AVE reference.

A recent study has suggested that the underlying processes of early vMMN reflect the neuronal refractory effect, while vMMN reflects the memory-comparison-based change detection effect (Kimura et al., 2009). Supporting this issue, there is evidence that the earlier vMMN components could be evoked under task-irrelevant stimuli, representing an automatic change detection mechanism (Astikainen and Hietanen, 2009; Maekawa et al., 2012). The results of the current study indicated a functional difference in automatic detection of changes in facial expression in schizophrenic patients.

Importantly, the vMMNs were significantly reduced in patients with schizophrenia compared to healthy controls, indicating the dysfunction of processing task-irrelevant facial expressions, when happy expressions acted as mismatch stimuli. Csukly et al. (2013) investigated the abnormality in the vMMN elicited by unexpected facial expressions in patients with schizophrenia, and found that mismatch responses to both fearful and happy emotional faces were significantly impaired in patients compared to age-matched controls. Although the conclusion of the Csukly et al. (2013) study is similar to that of the present study, there are several methodological differences. For instance, in this study, only paranoid and undifferentiated schizophrenic patients with emotional abnormity were recruited to more reliably investigate the processes underlying facial recognition.

It should be noted that emotional recognition was not required in the present study. Csukly et al. (2013) proposed that processing deficits of emotion might mediate the association between automatic information processing deficits in the daily lives of people with schizophrenia (Csukly et al., 2013). However, this issue needs further investigation. In addition, schematic emotional faces were used as experimental stimuli to minimize the variations of actual faces, including low processing-level facial features, as well as the possibility of gender effects. Previous findings have indicated that schematic faces may be useful for clinical study and application because of their simplicity compared to actual human faces (Wright et al., 2002). Although schematic emotional faces have been used in several studies (Chang et al., 2010; Xu et al., 2013) and similar vMMN results have been reported with real faces, it is necessary to use real faces to further investigate this issue.

In addition, the current study also revealed that the sad vMMN was significantly larger in the right than the left temporo-occipital area in schizophrenic patients. Because previous neuropsychological studies have suggested that the right hemisphere is relatively superior to the left in the perception of facial expression (Mandal and Singh, 1990; Borod, 1992; Mandal et al., 1993), especially negative ones (Davidson et al., 1990; Mandal et al., 1991), these evidences imply that automatically processing of negative facial emotion might be impaired in schizophrenic patients with dominant right hemispheres.

### Choice of reference in clinical ERP study

This study systematically investigated the face-expression neurophysiological markers in people with diagnosis of schizophrenia and healthy controls by comparing AVE and REST referencing methods. As a commonly recommended reference, AVE is conducted by averaging all the scalp electrodes. However, recent studies show that REST is more reliable with low-density montage (Liu et al., 2015; Yao, 2017). Considering the time costs and operational difficulties, most of the clinical studies that aimed at finding a reliable and effective biomarker to distinguish neuropsychiatric patients from the healthy population prefer a low-density montage design. In the current study, both AVE and REST methods could effectively distinguish the facial expression evoked ERPs and MMNs between schizophrenic patients and healthy controls in our low-density montage design, suggesting REST is an appropriate approach in clinical neurophysiological studies, which could be applied to large populations.

**Figure 7:**
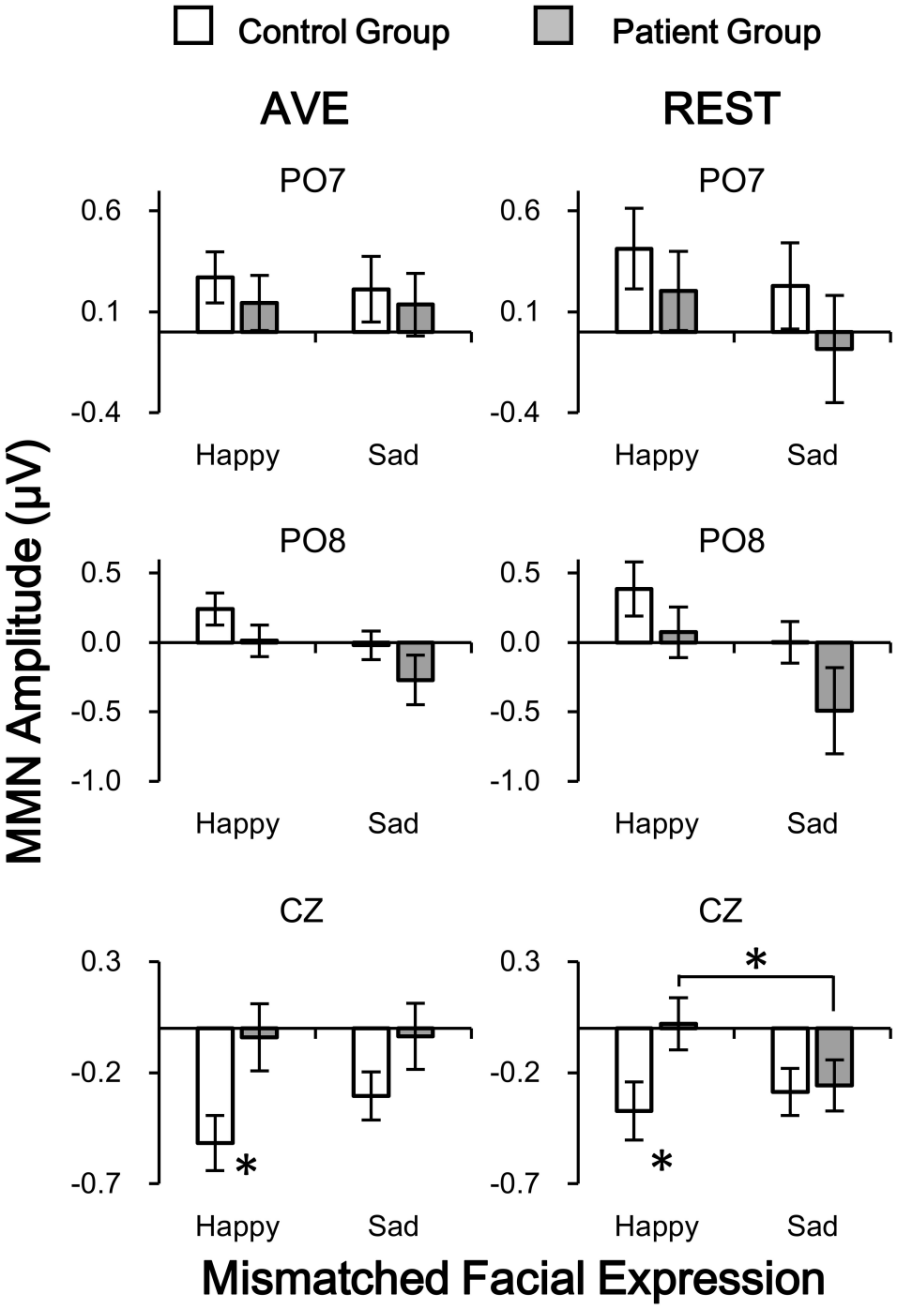
Comparisons of vMMN amplitudes in patient and control groups in temporo-occipital electrodes (PO7 and PO8) and central electrode (CZ) using two reference-modes (AVE and REST). White bars, control group; Gray bars, patient group. AVE, average reference; REST, reference electrode standardization technique. ^*^p < 0.05.

An interesting result from the current study was the finding that only the REST-obtained vMMN, but not the AVE-obtained vMMN revealed a significant difference between happy and sad mismatch stimuli in schizophrenic patients, but not in healthy controls (Figure 7). Critically, this finding dose not directly suggest that REST is superior to AVE. Approximately reconstructing a point far away from all the scalp electrodes, REST was suggested to be a neutral reference (Yao, 2001; Zhai and Yao, 2004; Liu et al., 2015; Chella et al., 2016). Therefore, REST usually achieves more objective results, which could possibly flip the significance of a result from the other references including AVE (Tian and Yao, 2013). Although the main purpose of the clinical ERP studies was to reveal neurophysiological difference between patients and controls, previous results obtained with a non-zero reference such as AVE need more confirmatory evidence, and so we recommend applications of REST in neurophysiological studies of neuropsychiatric disorders in the future.

## Author contributions

Each of the authors, SS, HL, YN, JR, RH, ZW, RH, JZ, QW, and YZ designed the study and wrote the protocol. SS, HL, and YZ performed the experiments. QW wrote the first draft of the manuscript. All authors contributed to and have approved the final manuscript.

### Conflict of interest statement

The authors declare that the research was conducted in the absence of any commercial or financial relationships that could be construed as a potential conflict of interest. The reviewer DY and handling Editor declared their shared affiliation, and the handling Editor states that the process nevertheless met the standards of a fair and objective review.
